# Experimental observation of gray whale skull vibrations amplified in the bony hearing complex

**DOI:** 10.1038/s41598-025-98100-1

**Published:** 2025-04-23

**Authors:** Margaret A. Morris, Petr Krysl, John A. Hildebrand, Ted W. Cranford

**Affiliations:** 1 Scripps Institution of Oceanography, University of California, San Diego, 9500 Gilman Drive MC:0205, La Jolla, CA 92093-0205 USA; 2 Department of Structural Engineering, University of California, San Diego, 9500 Gilman Drive MC:0085, La Jolla, CA 92093-0085 USA; 3 Department of Biology, San Diego State University, 5500 Campanile Drive, San Diego, CA 92182 USA

**Keywords:** Bioacoustics, Vibroacoustics, Mysticete, Gray Whale, Hearing, Biomechanics, Marine biology

## Abstract

Mysticete whales have bilateral bony ear complexes (tympanoperiotic complexes) that amplify low frequency vibrations in proximity to their vocalization ranges. Understanding the functional mechanics would enable precise predictions of mysticete hearing sensitivity, which is currently unknown. We conducted experiments on a juvenile and an adult gray whale skull from deceased animals to measure the vibrational dynamics between the tympanic bullae and the skull. Relative motions between bullae and skull indicate sound transfer to the inner ear. For the juvenile, assessments were performed on (1) a 3D-printed plastic skull-replica, (2) the original skull after much of the soft tissue had been removed by dissection, and (3) the denuded skull after hydrogen peroxide was used to erode the remaining soft tissues. We excited vibrations in the juvenile skull underwater by projecting sound in a test pool, ranging from 170–1000 Hz. Additionally, we measured in-air vibrations of the plastic, denuded, and adult skulls using a mechanical shaker to drive vibrations anteroposteriorly (rostrum-to-tail) from 150–1000 Hz. The results consistently showed amplification of vibrations at the tympanic bullae compared to the base of the skull, demonstrating a mechanism by which low-frequency sound is transferred from the environment, through the skull, to the inner ear.

## Introduction

Understanding the hearing sensitivity of mysticetes (baleen whales) is needed to decipher their acoustic behavior and to evaluate their vulnerability to anthropogenic ocean noise^1,20,21^. Conducting controlled experiments on live animals in a laboratory setting poses significant challenges because mysticetes are among the largest animals on Earth. In general, they vocalize at low frequencies with long wavelengths^18^. Gray whales (*Eschrichtius robustus*), the focus of this paper, produce sounds ranging from below 20 to 4000 Hz^2,8,16^, with wavelengths from $<1$ m up to 75 m at the lower end of this range. Although passive acoustic monitoring has been used to study mysticetes^10^, it is important to recognize that their vocalization frequencies may not correspond directly to their hearing capabilities^9^. Hearing mechanisms evolve under a variety of selective pressures that include but are not limited to: acoustic features of the environment; sounds from conspecifics, predators, or prey; the need to maintain contact with others in the the social group or to communicate valuable information for things like the location of mates or food patches across large expanses of the ocean. Behavioral studies in the animals’ natural settings can provide insight into the sounds that they can hear, but it does not reveal the full range of audible sounds. A more cost-effective and physics-based approach is to treat the whale’s anatomy as a mechanical system, for instance, by modeling using anatomic geometry obtained from CT scans of deceased mysticetes^4,11,14,22^. Simulating the whale’s anatomical response to an external stimulus can help resolve the underlying biomechanics of mysticete hearing. This modeling approach has proven successful in improving understanding the hearing mechanisms of odontocetes (toothed whales)^5–7,13^. Once refined, such models could produce synthetic hearing sensitivity curves^4^ for a variety of species.

**Fig. 1:**
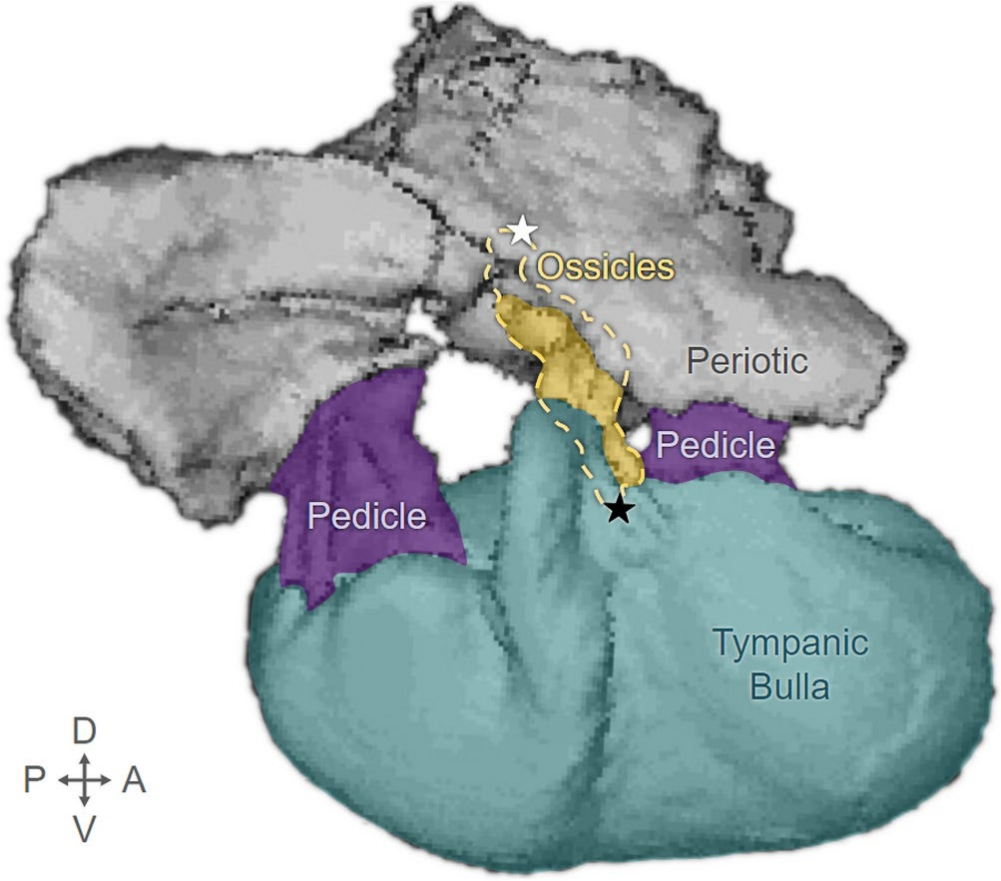
Components of the gray whale tympanoperiotic complex (TPC) highlighted on a CT reconstruction of the right TPC (Specimen: SDNHM 25307). Orientation directions are shown in the lower left corner (D = dorsal, A = anterior, V = ventral, P = posterior). Gray: periotic bone - embedded in the skull. Teal: tympanic bulla - suspended from the periotic by two pedicles (Purple). Yellow dashes: outline of the ossicular chain. Solid Yellow: part of the ossicular chain not obscured by the tympanic bulla or periotic. Black star: fused junction between the malleus (first ossicular element) and the tympanic bulla. White star: location of the oval window, the interface that allows the stapes footplate (last ossicle) to push on the fluid channels in the cochlea of the inner ear.

This study is an integral part of an ongoing effort to understand the sound reception mechanisms in mysticetes^3,4,17^. Our goal in this paper is to collect experimental measurements that can be used to validate biomechanical modeling. We focus on a mechanism by which low-frequency sounds are transferred from the environment, through the skull, into the inner ear. Within the mysticete bony ear complex (tympanoperiotic complex or TPC), the inner ear is housed within the periotic bone, which is anchored to the skull. One end of the ossicular chain contacts the inner ear at the oval window, and the other end is fused to the tympanic bulla (in land mammals this end is connected to the tympanic membrane). The tympanic bulla is suspended from the periotic by two thin, flexible bones (pedicles), allowing it to swing and twist relative to the periotic (Fig. 1). Because one end of the ossicular chain is fused to the tympanic bulla and the other end contacts the inner ear fixed in the periotic bone, relative motions between the tympanic bulla and the periotic bone indicate actions of the ossicular chain that can deliver impetus to the cochlear fluid, resulting in a perception of hearing. Finite element models indicate that vibrations received by the mysticete skull lead to amplified motions of the tympanic bullae at various natural (resonant modes of vibration) frequencies^3,4^. These findings underscore the important role that skull vibrations play in sound reception and transfer to the inner ear.

In the present study, we used the gray whale as an example of a non-rorqual mysticete to investigate whether vibrations excited in the skull of the whale were amplified in the tympanic bullae. Our experimental design involved measuring the relative vibration between the tympanic bullae and the base of a gray whale skull. We did not attempt measurements directly on the ossicular chain partly because it is tucked behind the bulla and also because the ossicular chain components do not have a surface large and flat enough for secure sensor attachment. Additionally, each bulla’s mass is large compared to the sensor mass, so its vibrations should not have been affected by the added mass of the sensor as much as the less-massive ossicular chain components may have been. These experiments were conducted on a juvenile gray whale in three forms of the same anatomic geometry and on one adult gray whale.

**Fig. 2:**
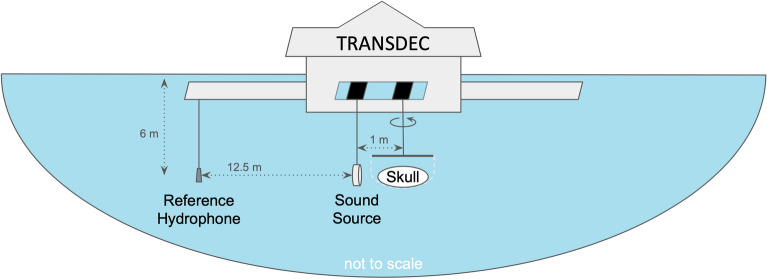
Schematic of underwater test arrangement at TRANSDEC. The skull and sound source were each lowered halfway down the water-column (5.5–6 m depth). The projector was mounted on a pole in a fixed position, while the skull hung in a net suspended from a pole that was able to rotate to a specified angle relative to the transducer. The source to skull centroid separation was 1 m, putting the tip of the rostrum within 1 m of the sound source. An H-52 reference hydrophone was suspended from a bridge 12.5 m from the sound source and at 6 m water depth.

**Fig. 3:**
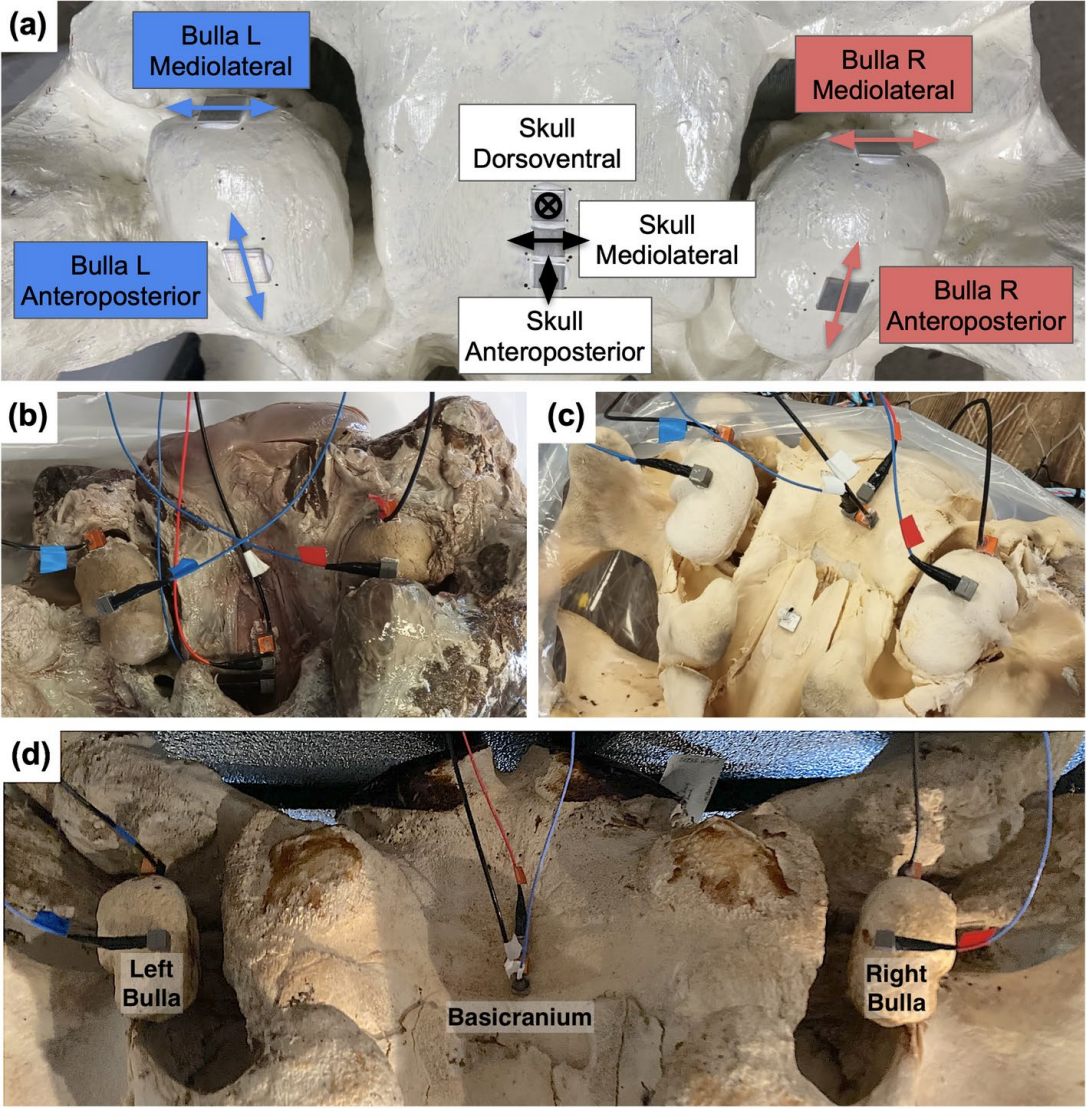
Accelerometer placement on skulls. (**a**) Plastic juvenile skull with aluminum plates attached to the plastic with *J-B Weld MarineWeld* epoxy. During experiments, accelerometers were superglued to the aluminum plates. Arrows show direction of measurement, and annotations describe the measurement that takes place at each location. (**b**) Sensors placed on natural juvenile skull. Accelerometers were superglued to the aluminum plates. In this case, the skull sensors are placed further forward on the skull to avoid remaining soft tissue. (**c**) Sensors on denuded juvenile skull. (**d**) Sensors on adult skull. In cases (**c**) and (**d**), sensors were glued directly to the bone (without aluminum plates).

The juvenile skull geometry was represented by three different forms: a plastic replica of the skull produced by 3D printing from CT scans of the original specimen; the original natural skull, after much of the soft tissue, superficial to the skull, had been removed by dissection; and the denuded skull, after hydrogen peroxide was used to erode soft tissues within the cavities of the skull. We excited vibrations in the juvenile skull, both natural and plastic replica, submerged underwater by projecting sound from 1 meter anterior to the skull in the Navy’s TRANSDEC test pool (Fig. 2), focusing on the 170–1000 Hz frequency range (range was determined by equipment and testing facility limitations). Additionally, we measured in-air vibrations of the plastic replica skull, the denuded juvenile skull in both frozen and thawed conditions, and the adult skull. These measurements were made using a mechanical shaker to drive vibrations anteroposteriorly (rostrum-to-tail) in each skull between 150–1000 Hz. In all cases, seven uniaxial accelerometers were placed on the skull: two on each bulla and three on the basicranium (Fig. 3). Sensor placement and measurement direction were designed to capture motions of the natural vibration modes of the skull predicted by finite element models.

Several experimental configurations were designed to address the importance of skull integrity and skull geometry, the directionality of sound reception, and the effect of the vibration excitation method. Testing of the juvenile skull underwater in its three different forms relates to skull integrity, as the plastic skull is a solid piece, the natural skull maintains ligamentous connections between the bones, and the denuded skull has weakened connections between the bones. In a similar way, testing multiple forms of the juvenile skull and the adult skull in air illustrates the importance of structural integrity of the whole skull. The plastic skull represents an exaggerated form of the skull in which all of the pieces are fused together, while on the other extreme, several bones are loose or disconnected from each other in the denuded skull. The plastic skull was also used in both air and water to investigate whether amplification of vibrations in the bullae occurs for structures of the same geometry but of different material than the natural skull. Amplified vibrations of the bullae in the plastic skull would suggest that the skull geometry evolved to facilitate such amplification. We examine the directionality of sound reception underwater by rotating the skull in the incident sound field, thereby changing the orientation of the skull relative to the sound source. We also examine differences between motions of the left and right bullae within experiments. To test whether it is vibration of the skull as a whole that leads to amplified motion of the bullae, we conduct in-air experiments on the plastic juvenile skull, the frozen-denuded juvenile skull, and the adult skull, in which we excite vibrations through direct forcing of varied locations on the skull other than the bullae.

Tests on the plastic skull are also used to alleviate concerns about the experimental setup. One concern is that the skull in water was suspended in a net and held down by weights in some cases, and therefore experienced loading. Using the plastic skull, we tested the effect of loading different parts of the skull by changing its orientation on the ground and by supporting it on varied materials, either foam or concrete. Another concern is that the sound field in the water may be complicated by the test configuration. By comparing results from the plastic skull both in water and in air, we show that the skull vibrations in air and in water are consistent when considering the change in media.

Our observations revealed that regardless of whether vibrations were excited in the skull through ensonification in water or through mechanical forcing in air, there was a consistent amplification of the tympanic bullae’s vibrations relative to the basicranium, provided that connections between the skeletal components remained intact or were restored. The left and right tympanic bullae exhibited varying degrees of sound amplification across different frequency ranges, and the comparative amplitude of vibrations between the left and right bulla were influenced by the position of the sound source in the water. These findings collectively suggest that the skull plays a significant role in hearing sensitivity and directional sound reception in mysticetes and thus in sound source localization.

## Results and discussion

### Experiments in water

We present results as frequency response functions (FRFs), which are calculated as velocity amplitude divided by source pressure as a function of frequency. Due to interference patterns within the TRANSDEC test pool, it was difficult to assess the extent to which peaks in the resulting velocity/pressure curves were true features of the frequency response of the skull or artifacts caused by the test environment. Velocity curves were all smoothed to account for interference and to reveal prominent features of the velocity/pressure frequency response curves. Additionally, we present results as ratios of velocity on the bullae to velocity on the basicranium.

#### Plastic versus natural versus denuded

We present measurements on the juvenile gray whale skull ensonified in water at $0^\circ$ (head-on) for its three forms: plastic, natural, and denuded. For each case, we show the FRFs for the mediolateral and anteroposterior sensors separately.

**Fig. 4:**
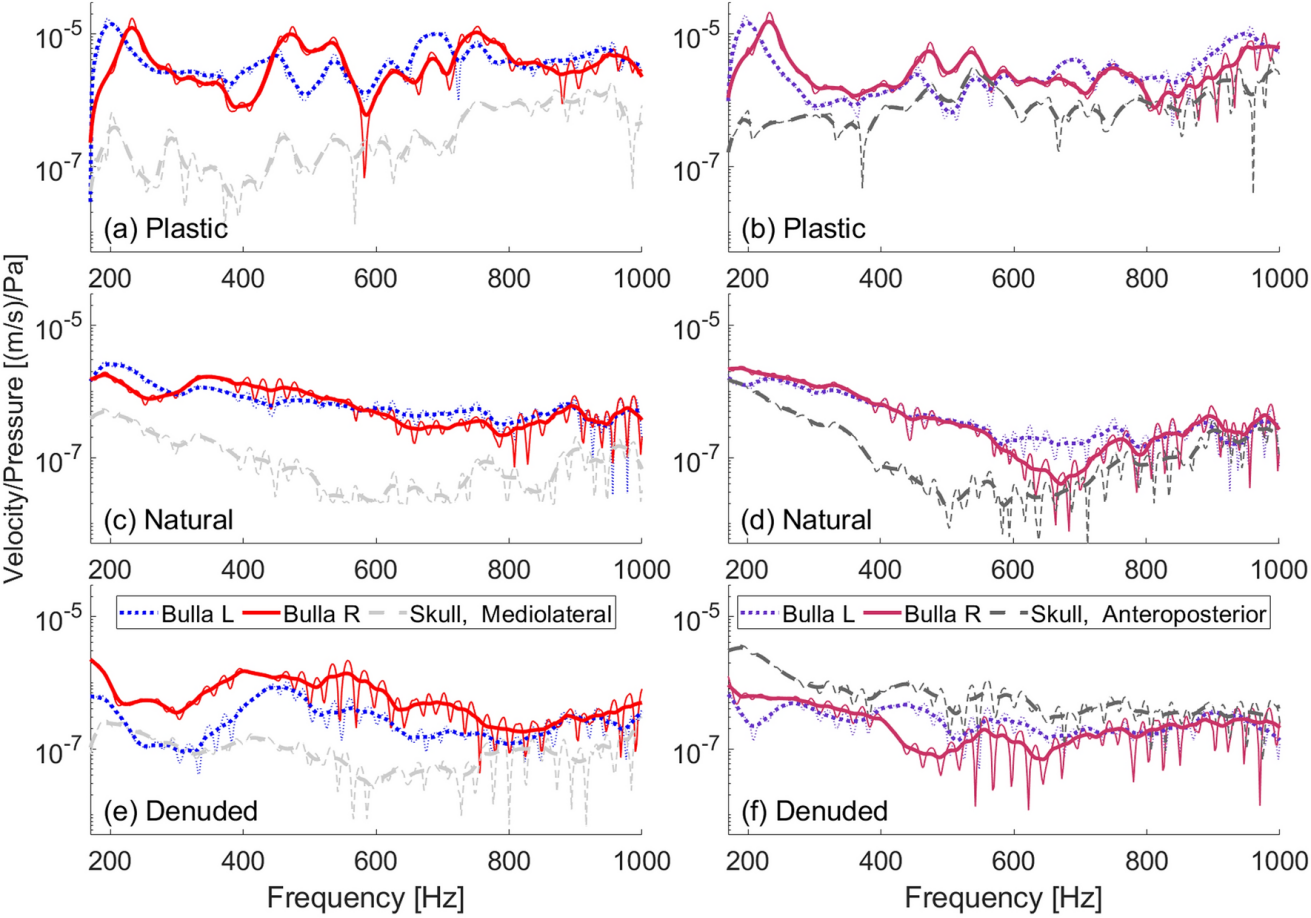
Frequency response functions obtained with a low-frequency underwater source ensonifying the juvenile skull in each of its 3 forms at $0^\circ$ (head-on) incidence. Smoothed curves are shown as thick lines atop the unsmoothed data. (**a**) Mediolateral velocity divided by incident pressure on the plastic skull. (**b**) Anteroposterior velocity divided by incident pressure on the plastic skull. (**c**) Mediolateral velocity divided by incident pressure on the natural skull. (**d**) Anteroposterior velocity divided by incident pressure on the natural skull. (**e**) Mediolateral velocity divided by incident pressure on the denuded skull. (**f**) Anteroposterior velocity divided by incident pressure on the denuded skull.

Among the three skull forms evaluated, the plastic skull had the highest velocity amplitudes and exhibited the most pronounced peaks (Fig. 4a,b). This enhanced vibrational response may be partially attributed to the uniform construction of the plastic skull, being one solid piece which facilitates efficient transmission of energy throughout its structure. This contrasts with the natural skull that is composed of a “jigsaw puzzle” of interlocking bony elements interconnected by ligaments (Fig. 4c,d), and the denuded skull (Fig. 4e,f) which exhibits further degradation in its connective structures. Importantly, the material differences between the plastic and the natural skulls inherently result in distinct vibrational spectra, so we would not expect the curves to match between the plastic and natural skulls. The importance of a solid skull-to-TPC connection is apparent in the denuded skull, whose left TPC was loose but whose right TPC was glued securely in place. These differences in attachment integrity led to significantly higher velocities for the right bulla compared with the left (Fig. 4e).

For the plastic skull in particular, different peak vibration frequencies were observed between the left and right bullae. When positioned head-on ($0^\circ$) relative to the sound source, the left bulla had greater vibrational responses at 200 and 690 Hz, in contrast to the right bulla, which exhibited vibration peaks at 230 and 750 Hz (Fig. 4). A similar, albeit less pronounced, asymmetrical pattern was observed in the natural skull’s response.

Mediolateral motions of the tympanic bullae demonstrated amplification over the basicranium throughout the full spectrum of frequencies tested (170–1000 Hz) in every scenario, with the exception of the frequency range 226–312 Hz for the left bulla of the denuded skull, where the TPC was not securely connected to the skull. The data for the denuded skull were the most erratic, evident in the unsmoothed curve (Fig. 4e,f). While the shape of the velocity ratio curve for the right bulla is similar between the denuded and natural skull, the higher peak is shifted in frequency (850 Hz in the natural skull and 900 Hz in the denuded) indicating that not all vibration modes are being excited in the same way. The anteroposterior motions of the bullae in both the plastic and natural skulls are amplified for most frequencies. In the denuded skull, however, the anteroposterior motions of the bullae are comparable in amplitude to those of the natural skull, but the anteroposterior motion of the basicranium is significantly increased. Also puzzling is that the anticipated reduction in motion for the less securely attached left bulla compared to the right was not observed.

#### Changing direction of incident sound

To investigate how the skull responds to sound coming from various directions underwater, we rotated the skull in the horizontal plane up to $45^\circ$ in both clockwise and counterclockwise directions. Clockwise rotations, denoted by positive angles, mean that sound approached from the right side of the skull (towards the right bulla), while counterclockwise, or negative angles, directed sound towards the left bulla, and $0^\circ$ is incident directly from the front, toward the rostrum.

**Fig. 5:**
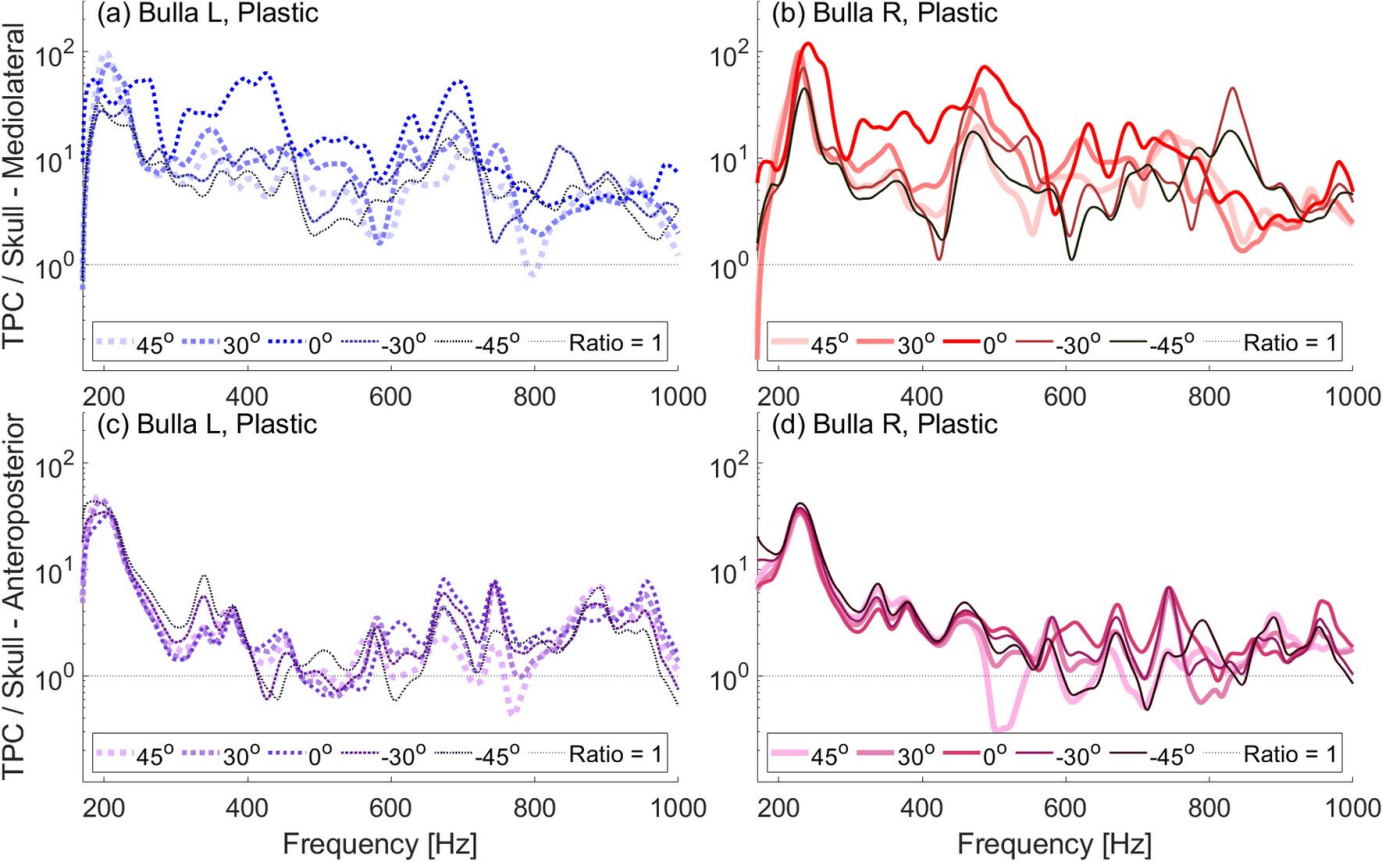
Frequency response ratios obtained with a low-frequency source ensonifying the plastic skull underwater between 170–1000 Hz at varying angles of incident ensonification ($0^\circ$ is head-on ensonification). (**a**) Ratio of left bulla to skull mediolateral velocity. (**b**) Ratio of right bulla to skull mediolateral velocity. (**c**) Ratio of left bulla to skull anteroposterior velocity. (**d**) Ratio of right bulla to skull anteroposterior velocity.

**Fig. 6:**
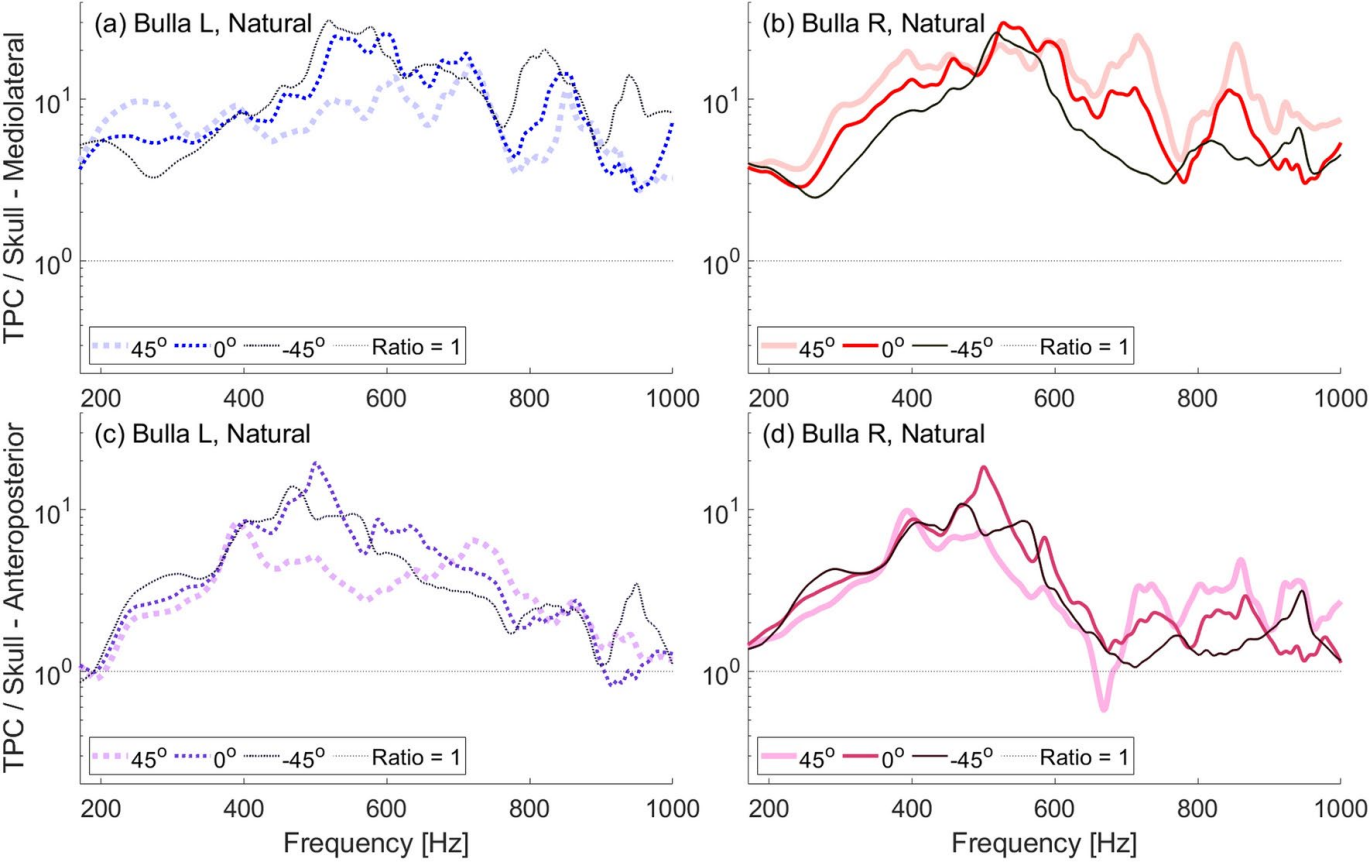
Frequency response ratios obtained with a low-frequency source ensonifying the natural skull underwater between 170–1000 Hz at varying angles of incident ensonification ($0^\circ$ is head-on ensonification). (**a**) Ratio of left bulla to skull mediolateral velocity. (**b**) Ratio of right bulla to skull mediolateral velocity. (**c**) Ratio of left bulla to skull anteroposterior velocity. (**d**) Ratio of right bulla to skull anteroposterior velocity.

**Fig. 7:**
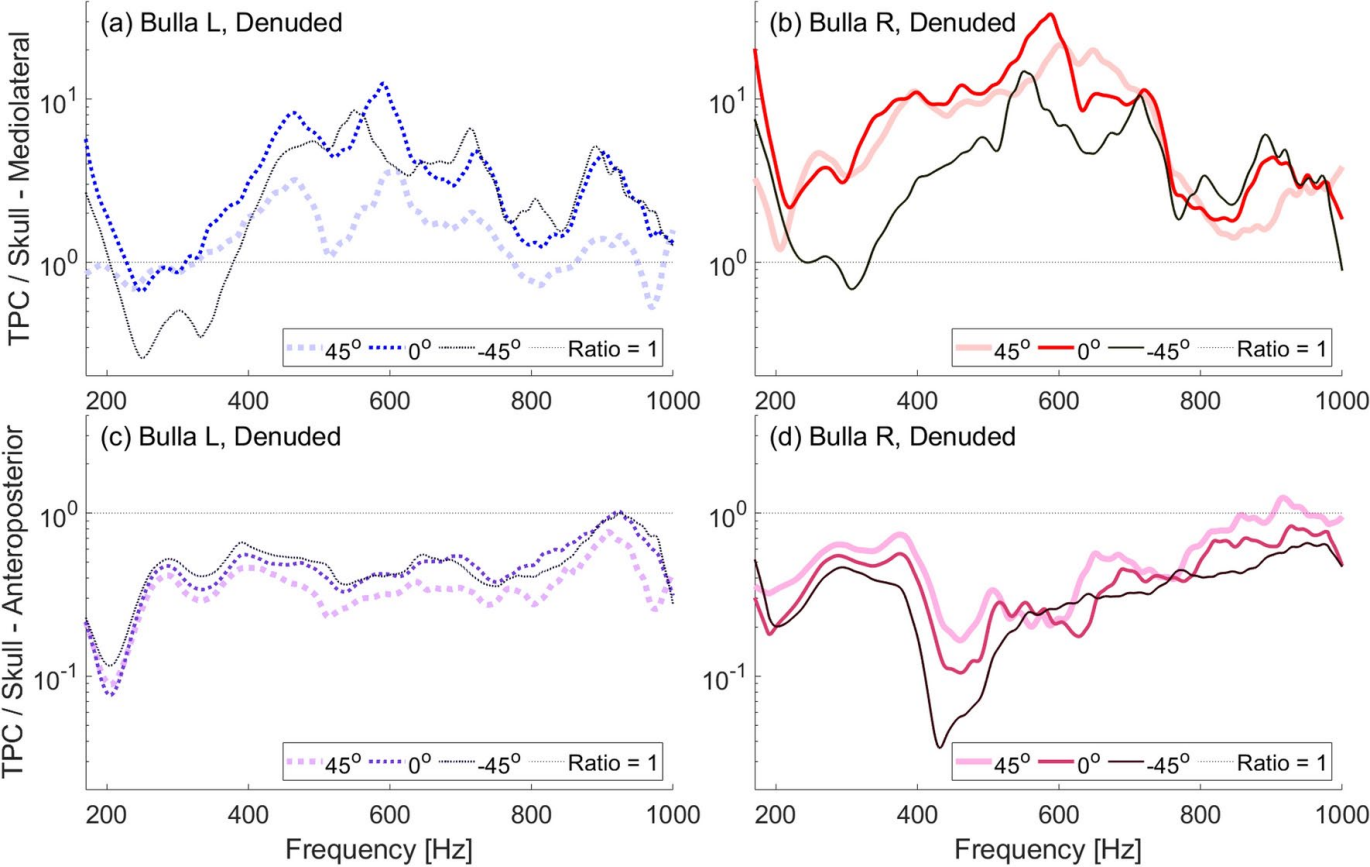
Frequency response ratios obtained with a low-frequency source ensonifying the denuded skull underwater between 170–1000 Hz at varying angles of incident ensonification ($0^\circ$ is head-on ensonification). (**a**) Ratio of left bulla to skull mediolateral velocity. (**b**) Ratio of right bulla to skull mediolateral velocity. (**c**) Ratio of left bulla to skull anteroposterior velocity. (**d**) Ratio of right bulla to skull anteroposterior velocity. Note that the y-axis scale of (**a**) and (**b**) is shifted compared to (**c**) and (**d**).

In our experiments, both the natural and denuded skulls show more amplified vibrations of the tympanic bulla closest to the incident sound. That is, at most frequencies in the range tested, the left bulla showed more amplification than the right when ensonified at $-45^\circ$ and the right is more amplified when ensonified at $45^\circ$ (Fig. 6, 7). This differs from the plastic skull in which, at most frequencies, direct incidence results in the highest amplification and off-axis incidence is similar whether it comes form the left or right directions (Fig. 5). The difference in plastic and natural skull response may indicate fine scale transmission between individual bones of the natural skull. While the plastic skull is a good approximation of the skull’s geometry, because it is a single homogenous piece, it is necessarily without some of the complexities of the natural skull.

In all cases, peaks are not always at the same frequencies between the mediolateral and anteroposterior directions nor are they at the same frequency when the skull is ensonified from different directions. For instance, the peak between 810–860 Hz in the mediolateral direction on the natural skull becomes lower in frequency as the angle of incidence decreases from $45^\circ$ to $0^\circ$ to $-45^\circ$ (Fig. 6a,b). This is evident in the denuded skull as well between about 890–930 Hz (Fig. 7a,b).

The shift in frequency of the peaks suggest that a change in the direction from which sound approaches the skull results in more than just changes in the amplitude of the sound received, but that different vibrational modes of the tympanic bullae may be excited by sound coming from different directions. In other words, the head’s anatomy may also work as a filter.

#### Decomposition of soft tissue within the natural skull

**Fig. 8:**
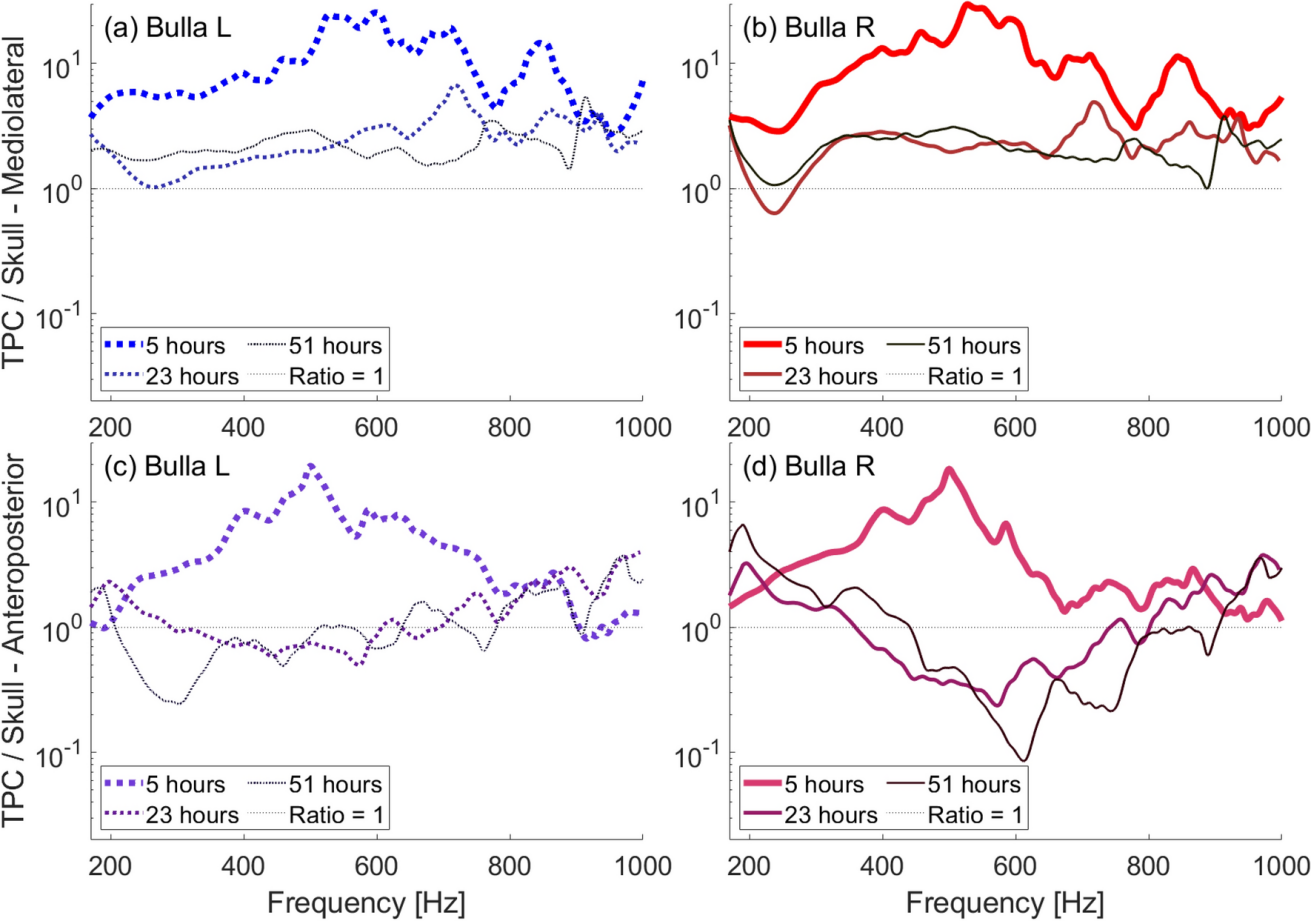
Frequency response ratios obtained with a low-frequency source underwater between 170–1000 Hz ensonifying the natural skull at $0^\circ$ (head-on) incidence for increasing lengths of time during which the skull was submerged in $80^\circ$F water. (**a**) Ratio of left bulla to skull mediolateral velocity. (**b**) Ratio of right bulla to skull mediolateral velocity. (**c**) Ratio of left bulla to skull anteroposterior velocity. (**d**) Ratio of right bulla to skull anteroposterior velocity.

Measurements on the natural skull were taken over the course of three days, when the skull had been in $80^\circ$F water, for 5 h, 23 h, and 51 h. On the second and third days, motions of the basicranium were noticeably higher, resulting in lower bulla to skull vibration ratios (Fig. 8). Vibrations in the dorsoventral direction were particularly high on those days. The increased vibration at the basicranium location was likely due to decomposition of soft tissues within the brain case. Decomposition gas bubbles were noticeably released by the skull on the second day of measurement. We attempted to release all of the gas by raising and lowering the skull in the water column; however, gas generated inside of the brain case would have been trapped just below the sensor location. Resonance of the trapped bubbles likely affected measurements on that part of the skull.

### Experiments in air

Due to the absence of a direct measure of the sound source level in our in-air experiments, we present our findings either as raw velocity measurements or as ratios of velocities. In addition, the in-air velocity curves were not smoothed due to the absence of multi-path interference observed for the in-water measurements.

#### Plastic versus denuded (frozen and thawed) juvenile versus adult skull

**Fig. 9:**
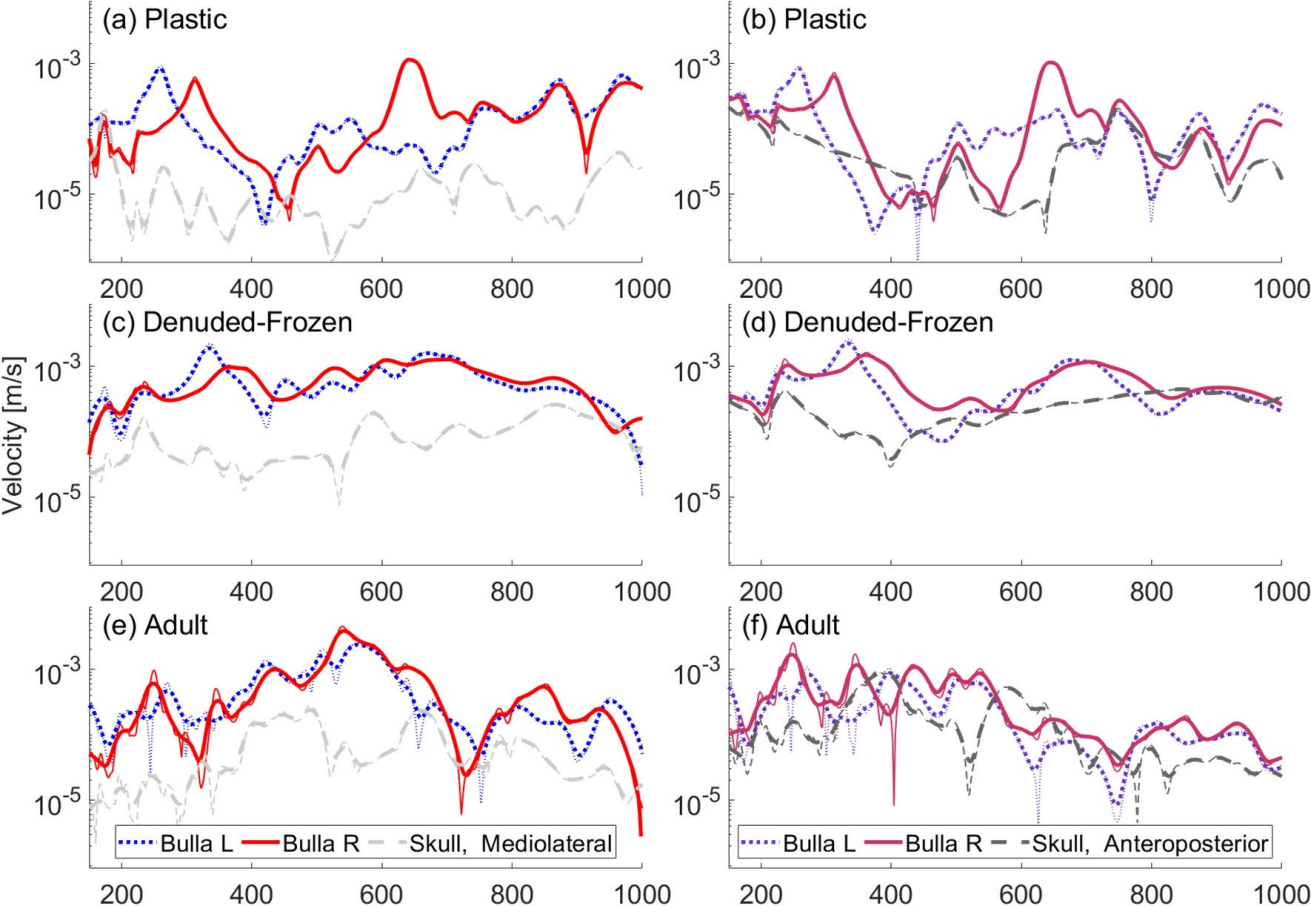
Frequency response functions obtained in air between 150–1000 Hz with a shaker driving the skull in air between the occipital condyles. (**a**) Mediolateral velocity on the plastic skull. (**b**) Anteroposterior velocity on the plastic skull. (**c**) Mediolateral velocity on the frozen denuded skull. (**d**) Anteroposterior velocity on the frozen denuded skull. (**e**) Mediolateral velocity on the adult skull. (**f**) Anteroposterior velocity on the adult skull.

We present measurements on the juvenile gray whale skull in its plastic and denuded state (both frozen and thawed conditions), in addition to measurements on the adult gray whale skull. Each experiment involved the application of anteroposterior vibrational forces to the skulls, using a shaker positioned between the occipital condyles (Fig. 14c–f). For each skull and condition, we show the FRFs for the mediolateral and anteroposterior sensors separately in Fig 9. The plastic juvenile skull exhibits the most pronounced vibrational peaks, followed by the adult skull, then the denuded-frozen juvenile skull. The denuded-thawed skull response (not shown) was smooth and steadily decreased with increasing frequency.

The observed variations in vibrational response align with the anticipated structural integrity of each skull condition. The plastic skull is one solid, fused piece, and it had the most distinct vibrational peaks. The adult skull’s sutures are mature, having fused the bones together with age and time, rendering it structurally solid. It also produced vibrational peaks. The denuded skull’s bones were only loosely held together when thawed, but had more solid connections due to ice and frost in their frozen state. Amplification of vibrations in the tympanic bullae relative to the basicranium is apparent in all cases except for the loose denuded-thawed case, suggesting that integrated skull components are required for vibrations to be transferred to the bullae.

The most significant peaks within each FRF curve, particularly those at lower frequencies, reveal shifts in frequency between the left and right bullae across different skull forms and conditions. For the plastic skull, the lowest peak velocity of the left bulla occurs at 260 Hz, contrasting with the right at 315 Hz. In the adult skull, the left bulla peaks at 272 Hz whereas the right peaks at 250 Hz. For the denuded-frozen skull, the left bulla shows peaks at 232 and 334 Hz, while the right’s are 242 and 362 Hz.

These variations in sound reception between the left and right bullae suggest a potential mechanism for directional hearing at low frequencies, where the asymmetry in sound reception could enhance the whale’s ability to discern sound direction. The separation of peaks also could increase the whale’s hearing sensitivity over a broader range of frequencies - the right bulla may receive sound more, where the left bulla receives sound less. Note that the left bulla peaks at a lower frequency in the juvenile skull and the right peaks at a lower frequency in the adult skull, indicating that the asymmetry is not decidedly in one way or the other for all gray whales.

#### Plastic skull tests of varied configurations

**Fig. 10:**
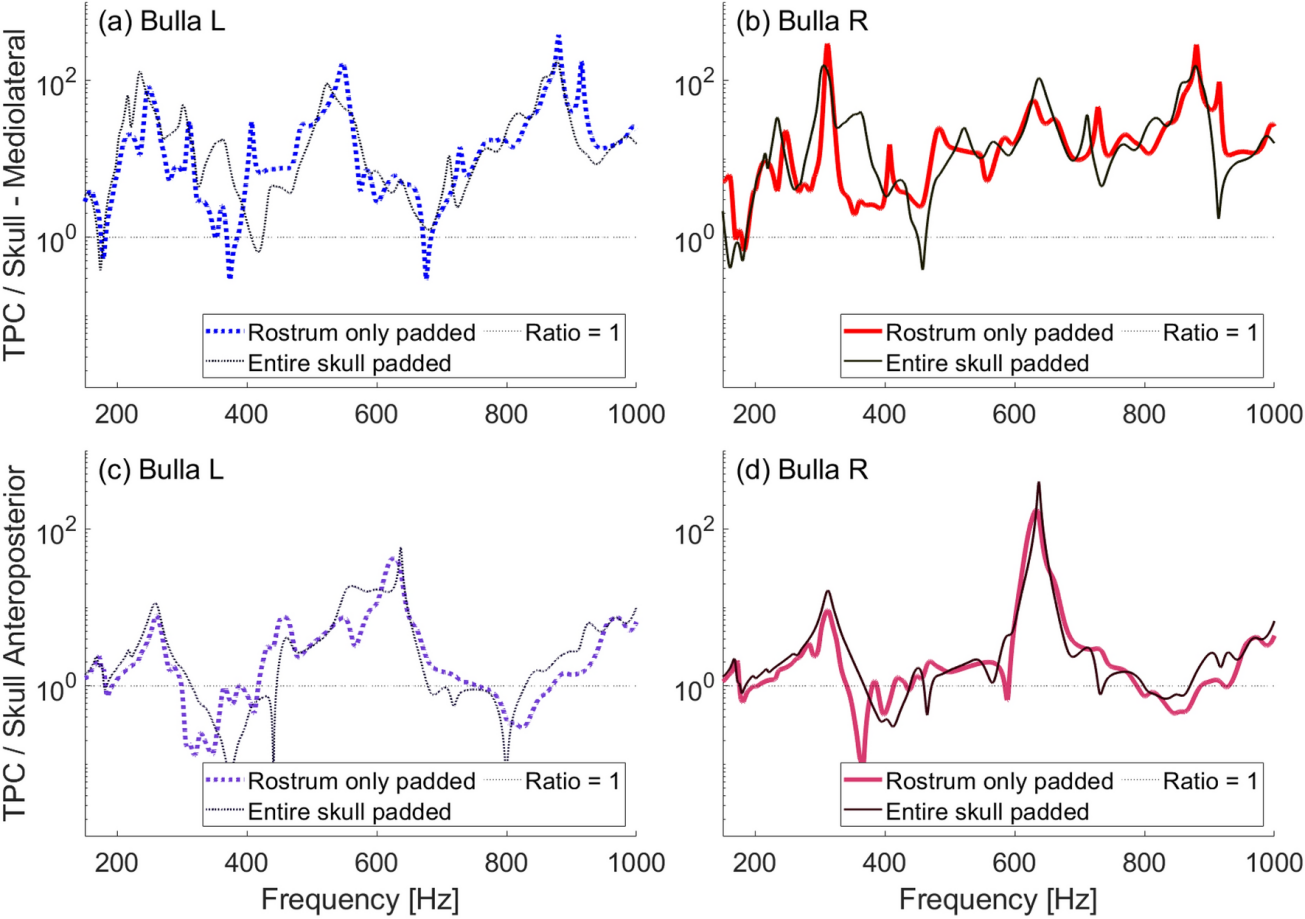
Frequency response ratios obtained in air between 150–1000 Hz with vibrations excited by a shaker on the occipital condyles of the plastic skull base. Different curves show different levels of padding between the floor and the skull, which was positioned ventral-side up. (**a**) Ratio of left bulla to skull mediolateral velocity. (**b**) Ratio of right bulla to skull mediolateral velocity. (**c**) Ratio of left bulla to skull anteroposterior velocity. (**d**) Ratio of right bulla to skull anteroposterior velocity.

To understand how skull positioning and loading may affect experimental outcomes, we ran a set of in-air experiments on the plastic skull in which we varied the position of the skull on the concrete ground and the amount of padding beneath it. Fig 10 presents velocity ratios for the plastic skull resting ventral-side-up with either the entire skull resting on padding, or the rostrum only on padding. These variations showed negligible differences in their vibrational response.

**Fig. 11:**
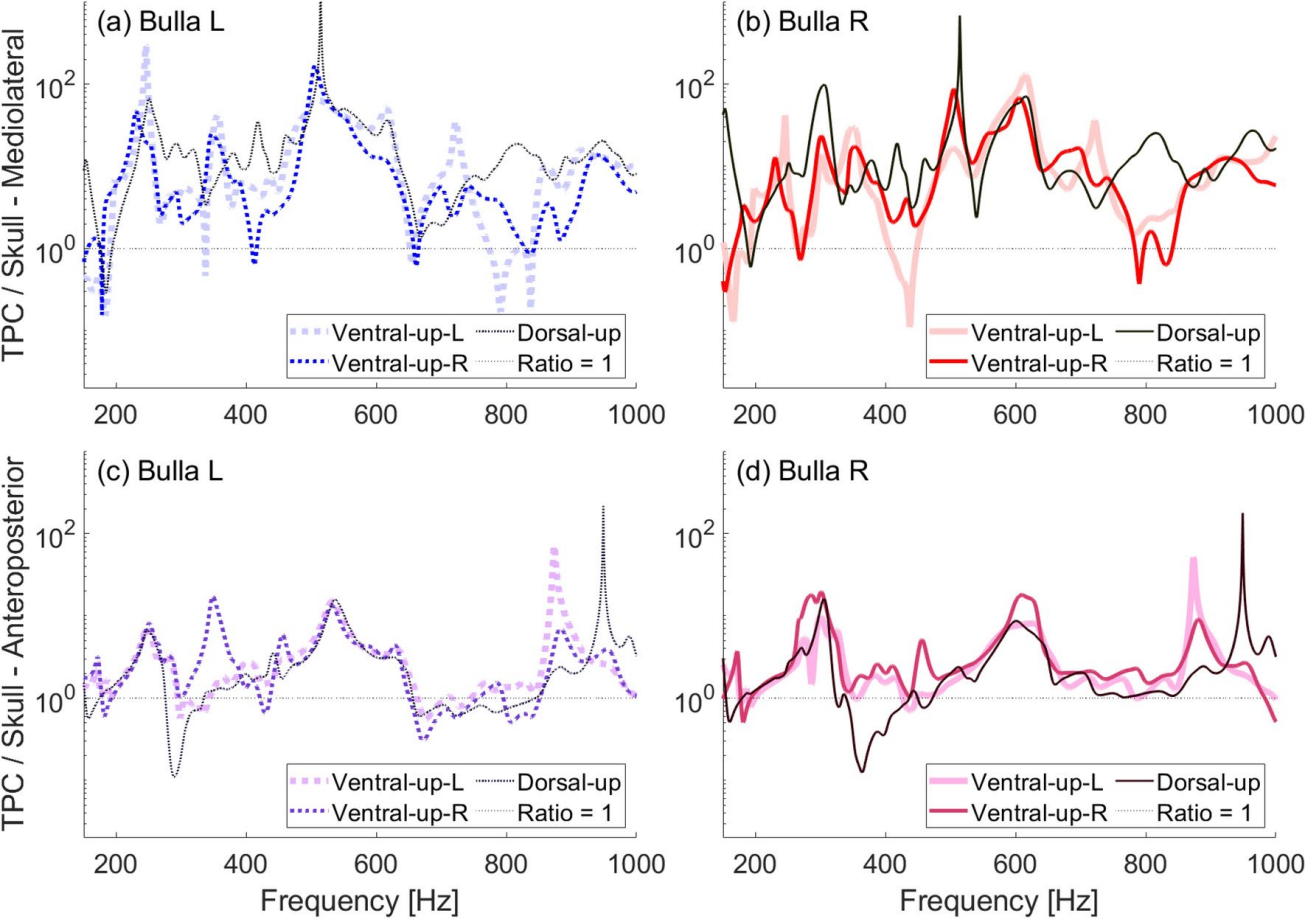
Frequency response ratios obtained in air between 150–1000 Hz with vibrations excited by a shaker at the plastic juvenile skull rostrum. Different curves show different positioning of the skull on the concrete ground. Ventral-up-L and ventral-up-R refer to the skull’s tilt when positioned ventral-side up: L means tilted toward the left bulla, while R means tilted toward the right bulla. (**a**) Ratio of left bulla to skull mediolateral velocity. (**b**) Ratio of right bulla to skull mediolateral velocity. (**c**) Ratio of left bulla to skull anteroposterior velocity. (**d**) Ratio of right bulla to skull anteroposterior velocity.

Further experimentation involved altering the skull’s orientation while resting on a concrete surface. Fig. 11 shows velocity ratios for the plastic skull resting on a concrete surface ventral-side up and tilted toward the left bulla, ventral-side up and tilted toward the right bulla, and dorsal-side up. While changing the skull’s orientation had some effect, we note that amplification of vibrations in the tympanic bullae remained substantial across all tested positions and substrates. Certain peaks in amplification are relatively unaffected, particularly between 480–650 Hz, regardless of the skull’s positioning.

While amplified vibration of the tympanic bullae remains a consistent observation in our in-air experiments, varying the skull’s orientation - specifically comparing ventral-up to dorsal-up positions - reveals differences in the vibrational response, particularly at frequencies above 800 Hz. The loading that results from the skull’s contact with the concrete may affect how the skull vibrates as a whole, changing the skull’s resonant modes of vibration. These changes could have a direct impact on the TPC, affecting how the bullae move with respect to the periotic bone which is firmly attached to the skull, and changing which modes of vibration are excited in the bullae. So, while some of the peaks we see in the in-air experiments are affected by how the skull rests on the ground, amplification of vibrations in the bullae is a persistent feature.

#### Plastic skull tests of vibration excitation location

**Fig. 12:**
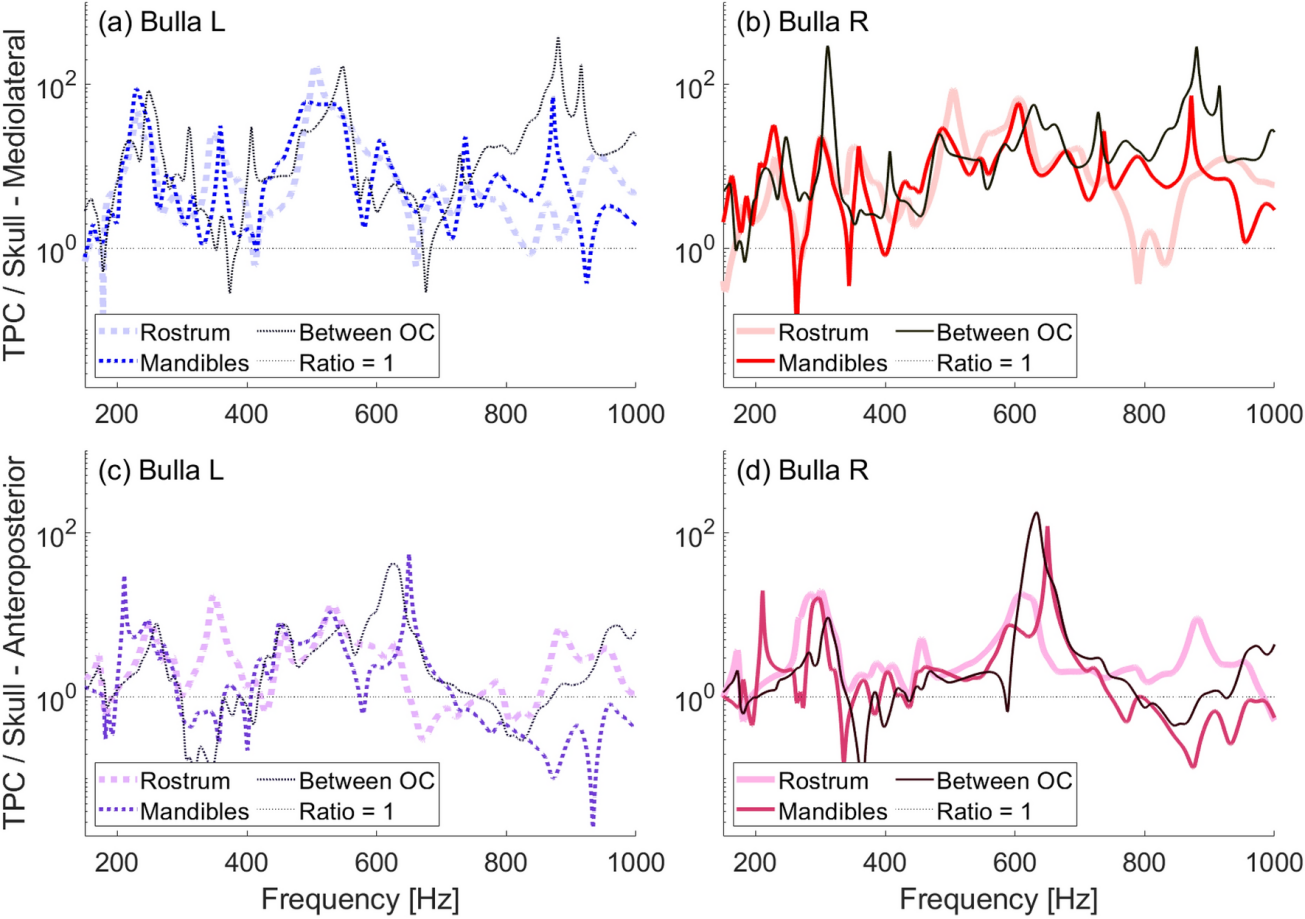
Frequency response ratios between 150–1000 Hz obtained in air with vibrations excited by a shaker at different points on the plastic juvenile skull. In all cases, the skull was resting ventral-side up, but the skull had a tilt to the right when driven at its rostrum. Legend indicates excitation location (OC = occipital condyles). (**a**) Ratio of left bulla to skull mediolateral velocity. (**b**) Ratio of right bulla to skull mediolateral velocity. (**c**) Ratio of left bulla to skull anteroposterior velocity. (**d**) Ratio of right bulla to skull anteroposterior velocity.

We conducted a set of experiments designed to investigate the effect of varying the point of vibrational stimulation on the skull, with the shaker driving vibrations anteroposteriorly into the skull at different stimulation locations on the skull. Fig. 12 compares the plastic skull excited at the tip of the rostrum, at the tip of the mandibles (equivalent to the location of the mandibular symphysis, a fibrocartilaginous joint between the mandibular rami in an actual skull), and the posterior end of the skull (between the occipital condyles). Across these varied stimulation locations, while some peaks occur at different frequencies, amplification the tympanic bullae’s vibrations is present across most frequencies tested.

### Water and air comparison

**Fig. 13:**
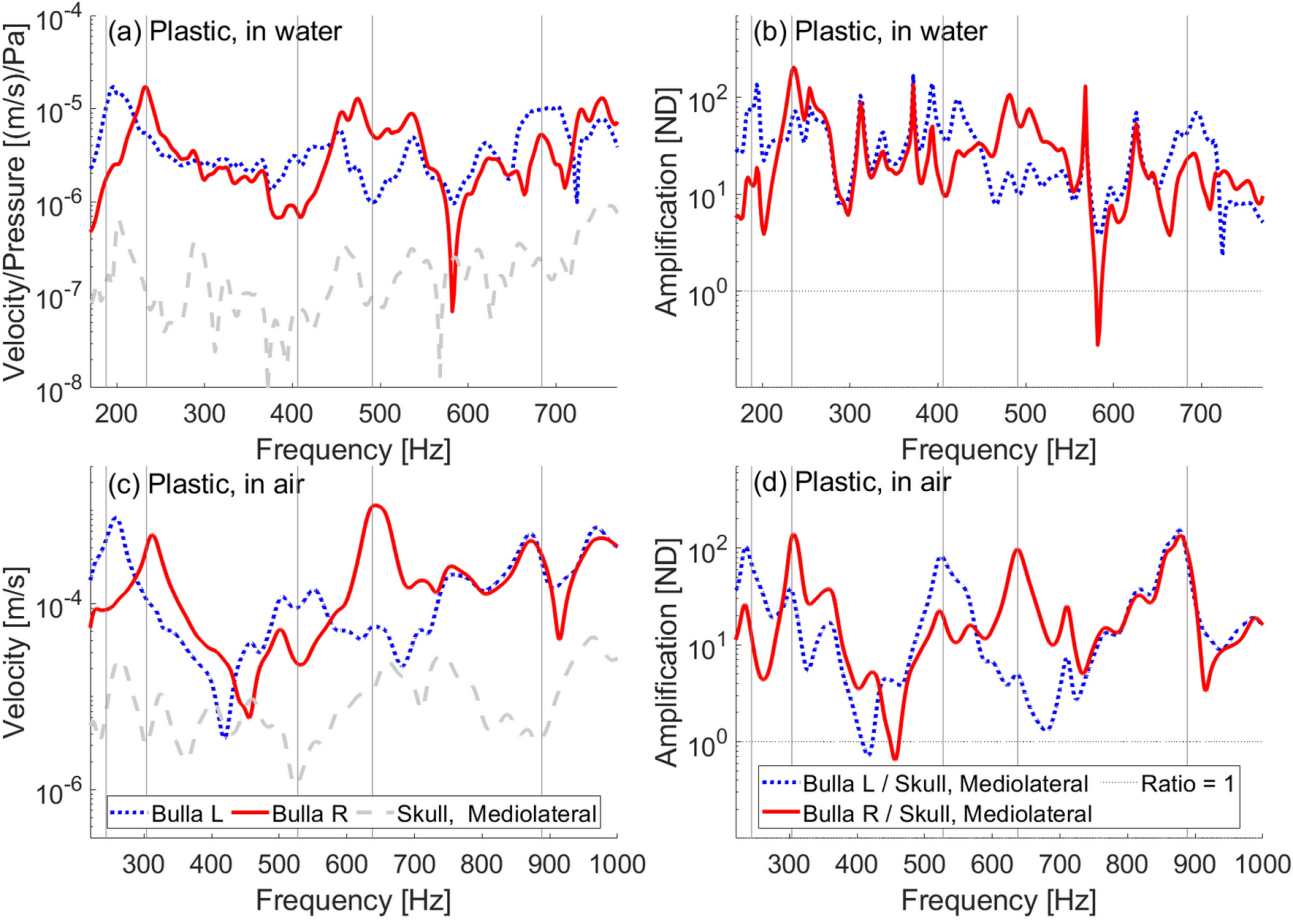
Comparison of in-water and in-air frequency response curves and ratios for the plastic juvenile skull. In-water results obtained with a low-frequency source ensonifying the plastic skull at $0^\circ$ (head-on). In air results obtained with vibrations excited by a shaker between the occipital condyles. (**a**) Mediolateral velocity divided by incident pressure on plastic skull in water between 170–770 Hz (**b**) Ratio of bullae to skull mediolateral velocity in water between 170–770 Hz (**c**) Mediolateral velocity on plastic skull in air between 220–1000 Hz (**d**) Ratio of bullae to skull mediolateral velocity in air between 220–1000 Hz. Note that the frequency axis is shifted by a factor of 0.77 between the in-water and in-air curves. Vertical bars are placed such that the frequency for the in-water plots is 0.77 times the frequency for the in-air plots.

For most of the frequency range tested, vibrations in the tympanic bullae are amplified in comparison to those at the skull base. This pattern persists both when vibrations were driven by a sound field enveloping the entire skull in water and when vibrations were driven anteroposteriorly through the in-air skull at a single location. When comparing the vibrational responses in water to those in air, the most prominent peaks in each tympanic bulla’s amplification occur in water at frequencies about 0.77 times those of their in-air counterparts (Fig. 13). This factor of 0.77 aligns with expectations of the impact of different media (air versus water) on the resonance frequencies, as the frequencies should be lower in water than in air by a constant factor due to increased loading on the skull. The constant shift implies that, when vibrations are excited in the skull at these frequencies, the tympanic bulla will move relative to the skull in a way that is dependent on the skull’s natural vibration modes. This further supports the notion that the bullae in water are not moved by the incident sound pressure, rather they are moved by the vibrating skull.

### Limitations and implications

The experiments presented here, which use accelerometers to directly measure the vibrations of a gray whale skull, are the first of their kind for any cetacean. Preparation and CT scanning of the specimen takes significant resources. Keeping the specimen well-preserved prior to experimentation is critical, as is made clear by the reduction in data quality as the skull decomposed (Sect. 2.1). A major goal of the study was to test whether the biomechanical model results can be supported by experiments. A validated model could ultimately provide accurate predictions of hearing sensitivity. A similar biomechanical model was validated for an odontocete specimen, a bottlenose dolphin, by audiogram^7^. There have so far not been reliable audiograms generated for mysticetes, which is where this study comes in. Such a complex study comes with limitations which we address here.

Some limitations arose from the experimental setup. The size of the TRANSDEC pool and equipment limitations restrict the frequencies used in the experiments to 170–1000 Hz, even though passive acoustics and behavioral studies suggest a broader acoustic range for gray whales. Because the TRANSDEC pool is a finite space, complex interactions of sound waves may have affected the acoustic field that insonified the skull. The skull was placed close to the sound source to increase signal-to-noise. At lower frequencies, this placed the skull within one acoustic wavelength of the sound source, but outside of the near-field (The near-field is described as $R^2/\lambda$, where *R* = transducer radius and $\lambda$ = wavelength. For 1 kHz, $\lambda =1.5$ m, for the M72-110 C-Bass transducer $R=\sim 0.15$ m, making the near-field $\sim$2 cm). One of the roles played by the plastic skull in these experiments was to independently test the hypothesis that skull vibrations lead to amplified bullae vibrations, to alleviate concerns regarding the experimental setup. We measured vibrations in the plastic skull in both air and water with comparable results (Sect. 2.3). By comparing results from the plastic skull both in water and in air, we show that the skull vibrations in air and in water are consistent when considering the change in media.

Another set of limitations relates to the specimens used in the study. A potential concern about the state of the juvenile skull tested is that the skull was not truly ‘natural’ in that some of the soft tissues on the skull were necessarily removed for sensor attachment. Measurements of the physical properties of cetacean fats and blubber indicate that their acoustical properties are similar to that of water^19^, so we do not expect that inclusion of the soft tissues would have made a significant difference in the experiment results.

Another consideration is that all of the in-water experiments were conducted on one juvenile gray whale skull (or a plastic replica of the same geometry). The smaller size of the juvenile skull, compared to a mature adult, made the already complex experiment practical. We think it reasonable to assume that juvenile and adult mysticetes would hear using the same mechanisms, especially considering evidence for precocial development within the tympanoperiotic complex^15^. An adult skull was tested in air to supplement the work done on the juvenile skull. As in the juvenile, amplification of the bullae’s vibrations compared to the skull was notable. We note that the adult skull’s bones had been solidly fused together with age, which suggests that hearing sensitivity may change throughout the whale’s life. We do not mean to imply that experiments like this one can determine hearing sensitivity curves or that a hearing sensitivity curve measured for a juvenile gray whale will apply to mysticetes of any age. Rather, we suggest that mysticetes are all likely to hear using similar mechanisms, and that one such mechanism involves sound being received through the skull and transferred to the inner ear by bone vibrations.

The fundamental experimental result from this study is that when the skull vibrates, the bullae vibrate with a greater amplitude than the skull. The result holds across specimens, media, and all experimental conditions. The only case in which motions of the bullae were not amplified was that of the denuded skull, in which the bones were not all connected. From this we conclude that the mysticete’s skull, when it vibrates, transfers sound to the bullae, and that the bullae cannot receive the same sound without the skull. This result was predicted by finite element models, which used the same methods previously validated through studies of odontocetes. Motion of the bulla is necessarily transferred to the ossicular chain because the malleus is rigidly fused to the bulla. How the motion transfers through the ossicular chain is not resolved by this experiment. With a validated biomechanical model, we will be able to make predictions about how the sound transfers through the ossicular chain. Further model development and experimentation will be needed to investigate how sound is transferred to the cochlear fluid and through the inner ear. This is an area for future study.

Another avenue for further investigation is the potential for directional hearing. In some frequency ranges, the left and right bullae vibrated with much different amplitudes when insonified by sound coming from different directions (Sect. 2.1), a result previously predicted by biomechanical models of a fin whale^3^. The left and right bullae also had their peak vibrations at slightly different frequencies when insonified head-on. The differing vibrational properties between the left and right bullae could provide a clue as to how the whales may be able to localize sound.

## Conclusion

We investigated the vibrational dynamics between the tympanic bullae and the skull base of gray whale skulls vibrating between 170–1000 Hz in water and 150–1000 Hz in air. Across most tested frequencies, vibrations of the tympanic bullae were amplified compared to those at the skull base, and therefore to the periotic which houses the inner ear. This pattern was seen both when vibrations were driven by a sound field enveloping the entire skull in water and when vibrations were driven anteroposteriorly through the in-air skull at a single location.

In both experimental media, the most pronounced peaks in tympanic bulla amplification occurred at comparable frequencies when in-air vs in-water loading was taken into account. This consistency suggests that when vibrations are excited in the skull, the tympanic bulla will move relative to the skull in a way that is dependent on the skull’s natural modes of vibration.

The vibrational response of the entire structure is characterized by frequency-dependent modes of vibration. The specific modes that become excited depend on the integrity of the connections between skull components. Amplification of the tympanic bulla’s vibration particularly depends on a secure connection between the periotic bone and the base of the skull. Through this connection, the periotic bone vibrates with the skull. Skull vibrations cause the dynamic components of each TPC, beginning with the suspension of the bulla by a pair of thin bony pedicles, to twist and swing in relation to the periotic bone. In this way, sound may be transferred from the environment, through the skull, to the dynamic components of the TPC (including the ossicular chain) which displace the fluids in the cochlea of the inner ear, contributing to the mechanisms of auditory perception in whales. Future work includes further experimentation alongside model development to investigate the mechanisms by which sound travels through the ossicular chain and through the inner ear.

A consistent observation across our experiments is that the left and right bullae exhibit their peak vibrations at different frequencies. They also show significant differences in vibration amplitude when the skull is insonified from varied angles. Further investigation is needed to fully understand the implications of these observations. This asymmetry may facilitate directional hearing at low frequencies. The separated peaks may also bolster the whale’s hearing sensitivity over a broader range of frequencies, taking advantage of the offset resonances between the left and right bullae.

In future work we hope to confirm and expand the present experiments on additional specimens of mysticete skulls. The limited number of samples was always a concern, but very difficult to address: suitable samples are exceedingly rare. An opportunity to repeat similar experiments would be crucially informed by the lessons learned in this work.

## Materials

### Juvenile Gray Whale skull

A juvenile gray whale head (LACM 97758, total length of the animal = 5.4 m) was obtained from the Natural History Museum of Los Angeles County and approved for study by Dr. Jorge Velez-Juarbe—Associate Curator, Mammalogy (Marine Mammals), and David Janiger—Collections Manager, Mammalogy (Marine Mammals). The specimen had been collected and transferred to a freezer soon after death to prevent tissue degradation. The specimen underwent X-ray computed tomography (CT) scanning in two phases, first intact with all tissues and subsequently after removal of soft tissues superficial to the skull. For the reader’s reference, the skull would fit within a box of dimensions: mediolateral 0.5 m, dorsoventral 0.43 m, anteroposterior 0.94 m.

The experiments described here were conducted on the juvenile whale head following soft tissue removal. For the first set of experiments, most of the soft tissue was removed except those in areas which were inaccessible with manual dissection tools. These areas included tissue within the brain case, venous plexes, and various muscles. Since some flesh remained, we refer to the skull in this form as the natural skull. For the second set of experiments, the rest of the soft tissue was removed by bathing the natural skull in a 12% hydrogen peroxide solution. After this treatment, the skull is referred to as the denuded skull. The chemical procedure removed the majority of the remaining soft tissue, but also compromised the ligaments, leading to loosened connections between cranial bones, notably between the tympanic bullae and the skull base. To mitigate this, the right TPC was reconnected to the skull with superglue and epoxy at two points: the posterior process of the periotic where it joins the squamosal, and the falciform process at the junction with the pterygoid. Conversely, the left TPC was intentionally left in a loosened state for comparison. The chemical procedure also weakened the ligamentous temporomandibular joints, so the mandibles were removed at that time.

### Plastic replica skull

**Fig. 14:**
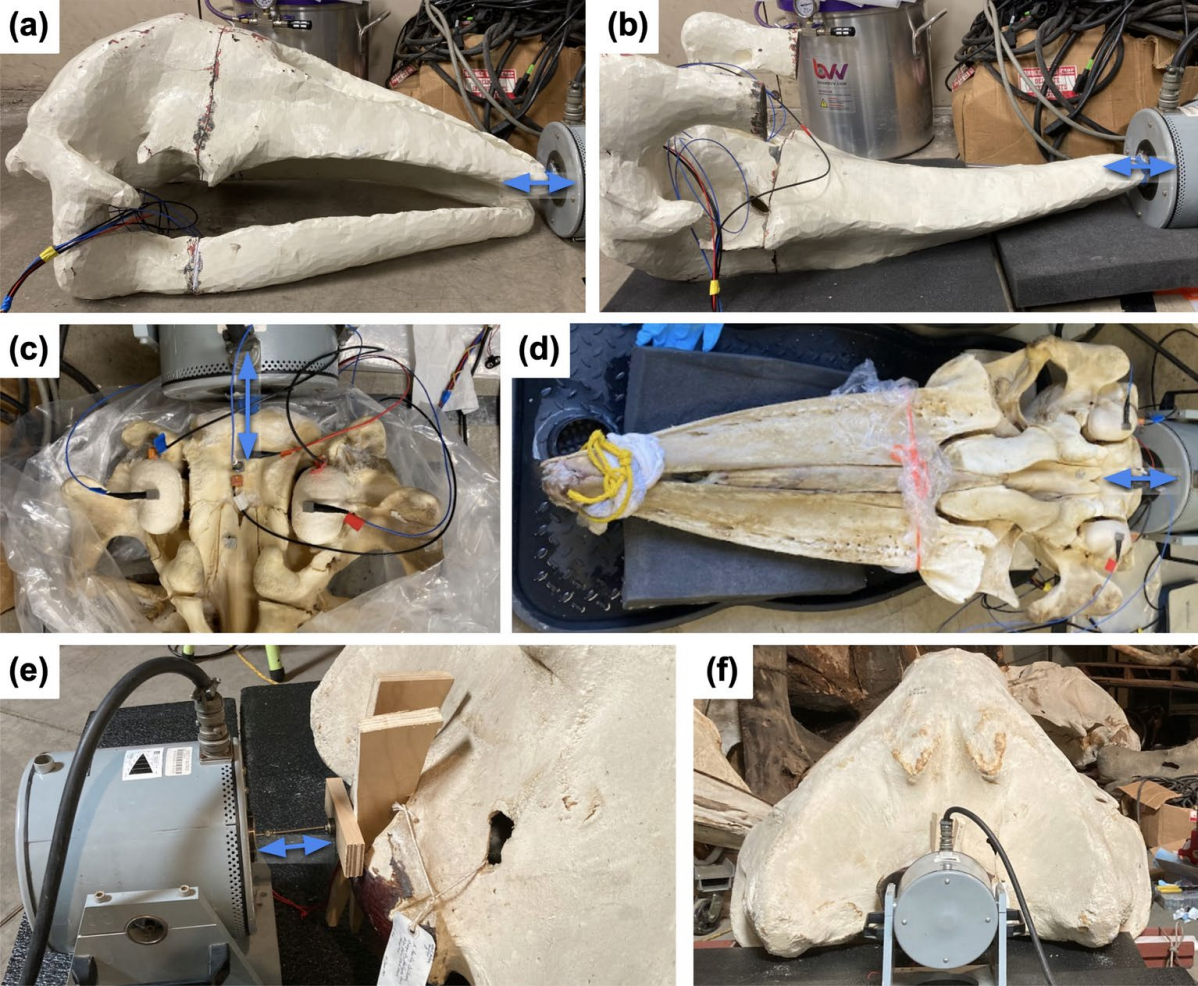
Setup of in-air measurements. (**a**) Plastic skull with mandibles, dorsal-side up, resting on a bare concrete. (**b**) Plastic skull without mandibles, ventral-side up, resting on one layer of foam. (**c**) Frozen denuded skull, ventral-side up, resting on one layer of foam. (**d**) Thawed denuded skull, ventral-side up, resting on a hard mat with one layer of foam beneath the rostrum. (**a**) and (**b**) show the rostrum pushed up against the vibrating plate of the shaker. (**c**) and (**d**) show the shaker plate positioned between the occipital condyles. In all juvenile-skull cases, the shaker was oriented to vibrate horizontally, pushing anteroposteriorly into the skull, and blue putty was wedged between the skull and vibrating plate to improve coupling. (**e**), (**f**): Adult gray whale skull, dorsal-side up, resting on stiff foam and museum cart. In this case, the shaker was fastened to the skull through the foramen magnum, between the occipital condyles. Accelerometers were glued to the bullae and skull as shown in Fig. 3.

A plastic replica of the juvenile gray whale skull was produced using 3D printing using solid ASA plastic (acrylonitrile styrene acrylate). This replica was based on the skull geometry from the CT scan of the gray whale head, capturing the anatomic geometry of the skull with high fidelity. Unlike the real skull, the plastic replica is a unified homogenous structure rather than a collection of individual bones with ligamentous connections. The fabrication of the replica involved arbitrarily dividing the skull’s volume into three segments: the base, the rostrum, and the anterior mandibles. The rostrum and mandibles were glued to the base with superglue and epoxy to make one solid cohesive unit (segment delineations are visible in Fig. 14a,b).

### Adult gray whale skull

A museum preparation of an adult gray whale skull (LACM 84202, condylobasal length = 252 cm), was tested at the Natural History Museum of Los Angeles County, Mammalogy Research Collections facility. The specimen, collected in 1989, was prepared so as to preserve the skull with both bullae intact. To aid our readers, we note that the adult skull was larger than the juvenile skull by approximately a factor of 1.6 in each direction.

### Sensors

To accurately measure motions of the tympanic bullae and the skull, we employed a set of seven uniaxial accelerometers comprised of three ASC 4221MF-002 accelerometers, three PCB 333B50 accelerometers, and one Dytran 3305A3 accelerometer. These sensors were selected for their high sensitivity across the frequency range of 103000 Hz (Manufacturer ratings were ASC: 138 mV/(m/s$^2$) $\pm 3$ dB for 01050 Hz, PCB: 102 mV/(m/s$^2$) $\pm 5\%$ for 0.53000 Hz, Dytran: 51 mV/(m/s$^2$) $\pm 5\%$ for 0.2–5000 Hz). Additionally, the lightweight nature of these sensors (ASC: 22 g, PCB: 7.5 g, Dytran: 3.7 g) minimized potential interference with the natural vibration patterns being measured. The use of three different sensor types was due to supply and budgetary constraints.

To ensure consistency across the data collected by these varied sensors, we conducted in-house calibrations. We calibrated all sensors between 100–3000 Hz at 1 Hz intervals. For each frequency, we used LabView software to generate a sinusoidal signal that activated an Unholtz-Dickie model 5PM shaker. Each sensor was securely attached to the shaker platform, oriented so that vibrations would be aligned vertically with its principal measurement axis. During the calibration process, an MTI 2000 fotonic sensor was employed to monitor the vertical displacement of the shaker platform. This displacement was subsequently converted to acceleration by multiplying it by $\omega ^2$. The fotonic sensor and accelerometers recorded data concurrently over a 1 s timeframe, and the amplitude was measured using an FFT method. The voltage readings from the accelerometers were then normalized against the acceleration measurements from the fotonic sensor, yielding the sensitivity as a function of frequency in V/(m/s$^2$). Across all sensor types, we observed that the calibrated sensitivity remained relatively stable for frequencies up to 1000 Hz, after which the sensitivity decreased as frequency increased. In the present study, the results are shown only between 150–1000 Hz.

## Methods

Our experimental methodology involved exciting vibrations of a known frequency within the skull and measuring the resultant acceleration at selected points on the skull. In water, the vibrations were excited through a submerged transducer, which created a sound field around the skull at the designated frequency. Conversely, in air a mechanical shaker was used to directly impart vibrations into the skull. The specifics of these experimental setups will be described in detail in other sections.

Experiments in water were performed on the juvenile skull in three different forms: the natural skull, the denuded skull, and the plastic replica. In-air tests were conducted on the plastic skull, the denuded skull while frozen, the denuded skull thawed, and the adult skull. In each experimental setup, two accelerometers were positioned on each bulla, and three additional sensors were placed on the basicranium (Fig. 3). The placement of the three sensors on the basicranium was designed to capture a comprehensive range of motion: one sensor was oriented to measure anteroposterior (rostrum-to-tail) motion, the second measured mediolaterally (left-to-right), and the third measured vibrations in the dorsoventral direction (normal to the surface of the basicranium). On each tympanic bulla, the sensors were arranged so that one accelerometer measured anteroposterior vibrations on the ventral keel of the tympanic bulla, and one measured mediolateral motions on the posterior apex of the bulla.

The placement of sensors on the tympanic bullae was informed by computational simulations^12^ that explored the vibrational behavior of these structures. The simulations showed that, for the fundamental vibration mode, the bullae exhibited a pendulum-like motion, swinging on the pedicles, giving the posterior apex significant mediolateral motion. In other vibrational modes, the bullae exhibit a twisting motion that produced significant anteroposterior motion on their ventral surfaces, alongside the mediolateral motion on the posterior end.

The locations of sensors on the skull were chosen to be between the left and right bullae. On experiments with the natural skull (Fig. 3b), we adjusted the placement of the skull sensors anteriorly, because a thin layer of tissue remained on the posterior section of the basicranium, preventing sensor attachment in the usual spot.

### Experiments in water

Our experiments in water consisted of exciting vibrations of a known frequency, using a submerged transducer to create a sound field surrounding the skull, and measuring the acceleration at selected points on the skull. These tests took place at the Naval Information Warfare Center (NIWC) Pacific’s Transducer Evaluation Center (TRANSDEC) facility in San Diego, CA, USA. The TRANSDEC pool is eye-shaped, spanning about 90 m by 60 m by 11.6 m deep at its center and holding approximately 23 million liters of fresh water. The pool was designed to be a low ambient noise transducer calibration and underwater acoustic test facility. All tests were conducted in the center of the TRANSDEC pool (Fig. 2).

The skull and sensors were lowered into the pool from the lab space, centered over the pool above the water. The skull was contained within a large-mesh monofilament net bag, itself suspended from two horizontal metal pipes which were fastened to a vertical pole. To counteract buoyancy, weights were used to keep the skull and net submerged. For the plastic skull, a 5 lb ($\sim$ 2.3 kg) weight was attached to the net, for the natural skull a 10 lb ($\sim$ 4.5 kg) weight was suspended from the rostrum and a 5 lb weight was wrapped around the mandibles, and for the denuded skull, two 5 lb weights were attached to the net. The denuded skull was held in a plastic bag inside of the net.

The pole holding the net bag with the skull inside was lowered into the water and set on a track that allowed the skull to move horizontally toward or away from the sound-emitting transducer, which hung from another pole mounted to the same track. The skull in the net and sound source were each lowered halfway down the water-column (5.5–6 m depth). The sound source was attached to a pole in a fixed location, while the skull hung from a pole that was capable of rotating to adjust the angle of incidence in the horizontal plane. The source and skull centroid separation was 1 m, putting the tip of the rostrum within 1 m of the sound source. Additionally, an H-52 reference hydrophone was suspended from a bridge 12.5 m from the sound source, in line with both the source and the skull, at a depth of 6–6.5 m.

Our experiments were conducted in the frequency range 170–1000 Hz, using a GeoSpectrum Technologies M72-110 C-Bass transducer. The low-frequency cutoff at 170 Hz was due to a limitation of the sound source’s operation while the high-frequency cutoff at 1000 Hz was due to decreased sensitivity of the accelerometers. Each experiment was run as a frequency sweep, stepping through the specified frequency range at 2 Hz intervals. Each frequency was projected tonally for 500 milliseconds at one-second intervals. Accelerometer data were recorded during each step for 700 ms.

We calculated the amplitude of acceleration at each frequency step by focusing on the steady-state portion of the recorded data employing a standard FFT method. Acceleration was then converted to velocity by dividing the angular frequency $\omega$. Source level was estimated using the manufacturer transmit voltage response curve combined with known drive voltages.

The outcomes of our experiments are represented as frequency response functions (FRFs), calculated as the ratio of velocity amplitude to source pressure amplitude as a function of frequency (in the interest of brevity we will omit “amplitude” in the rest of the text, but it will be implied in our results). Notably, all experimental data recorded in the TRANSDEC pool, including sound pressure level (SPL) and sensor velocity vs frequency curves, exhibited interference patterns. These patterns are indicative of constructive and destructive interference due to multi-path propagation within the pool. To reduce the chances of erroneous peaks being introduced to the FRFs by interference at the reference hydrophone location, the SPL curves were estimated from the manufacturer’s transmit voltage response curve. To further remove effects due to interference patterns in the TRANSDEC pool, we present results as ratios of velocities measured by different sensors. Noise introduced by the interference patterns are still apparent in the measured velocities and their ratios, but large-scale patterns can also be observed.

### Experiments in air

Our experiments in air used an Unholtz-Dickie model 5PM shaker to drive anteroposterior (rostrum-to-tail) motions in the skull at specified frequencies, and measured acceleration at the same points used in the water experiments (Fig. 3, 14c). Employing LabView as the signal generator, the shaker vibrated for 1 s, stepping from 150 to 1000 Hz in 1 Hz intervals. At each frequency step, accelerometer data were recorded for 1 s, allowing the amplitude of acceleration to be calculated. The data were then converted to velocity by dividing by the angular frequency $\omega$. Due to the lack of direct source level measurements in the air, results are presented as velocity ratios.

These experiments were conducted on the plastic skull replica, on the denuded skull in both frozen and thawed states, and on the adult skull. For the juvenile skull each iteration involved the shaker platform being coupled to the skull using blue putty between the occipital condyles (Fig 14c,d). For the adult gray whale, a more robust attachment was achieved by securing the shaker through the foramen magnum with a steel stinger, wooden plates and wedges (Fig 14e). Additional tests were conducted on the plastic skull under various configurations to assess how results were affected by the skull’s orientation and contact with the ground, the presence or absence of the mandibles, the padding beneath the skull, and the location of forcing by the shaker.

A complete list of in-air experimental configurations follows (* denotes configurations corresponding to Fig. 14).

**A: Plastic skull with mandibles**

- Driven at the tip of the rostrum

*Dorsal-side up, resting on bare concrete
- Ventral-side up, tilted toward left bulla, resting on bare concrete
- Ventral-side up, tilted toward right bulla, resting on bare concrete

- Driven at the tip of the mandibles

- Ventral-side up, resting on bare concrete

- Driven between the occipital condyles

- Ventral-side up, resting on concrete with one layer of foam beneath the rostrum
- Ventral-side up, resting on one layer of foam with an additional foam layer beneath the rostrum

**B: Plastic skull without mandibles**

- Driven from the tip of the rostrum

*Ventral-side up, resting on one layer of foam
- Ventral-side up, resting on bare concrete
- Dorsal-side up, resting on bare concrete

**C: Frozen denuded skull without mandibles**

- Driven between the occipital condyles

*Ventral-side up, resting on one layer of foam

**D: Thawed denuded skull without mandibles**

- Driven between the occipital condyles

*Ventral-side up, resting on hard mat with one layer of foam beneath rostrum
- Ventral-side up, resting on one layer of foam with an additional foam layer beneath the rostrum

**E: Adult skull without mandibles**

- Driven between the occipital condyles (through the foramen magnum)

*Dorsal-side up, resting on hard foam and museum T-cart.

## Data Availability

Acceleration vs frequency data from the experiments will be published on the Dryad repository as: Morris, Margaret (Forthcoming 2024). Recorded vibrations of gray whale skulls to study how vibrations in the skull are amplified in the bony hearing complex to facilitate low frequency hearing [Dataset]. Dryad. https://doi.org/10.5061/dryad.dbrv15f90.

## References

[1] Emily, C., Brandon, L. Southall, M. R., & Howard C. R. International policy, recommendations, actions and mitigation efforts of anthropogenic underwater noise. *Ocean Coast. Manag.* 202. 10.1016/j.ocecoaman.2020.105427 (2021).

[2] Crane, N. L., & Lashkari, K. Sound production of gray whales, *Eschrichtius robustus*, along their migration route: A new approach to signal analysis. *J. Acoustical Soc. Am.*, **100**(3), 1878–1886. 10.1121/1.416006 (1996). 10.1121/1.416006 8817910

[3] Cranford, T. W. & Krysl, P. Sound Paths, Cetaceans. *Encycl. Mar. Mammals* 901–904. 10.1016/B978-0-12-804327-1.00236-3 (2018).

[4] Cranford, T. W. & Krysl, P. Fin whale sound reception mechanisms: Skull vibration enables low-frequency hearing. *PLoS ONE* **10**(1), 10.1371/journal.pone.0116222 (2015). 10.1371/journal.pone.0116222 PMC4310601 25633412

[5] Cranford, T. W., Krysl, P. & Amundin, M. A new acoustic portal into the odontocete ear and vibrational analysis of the tympanoperiotic complex. *PLoS ONE*, **5**(8). 10.1371/journal.pone.0011927 (2010). 10.1371/journal.pone.0011927 PMC2915923 20694149

[6] Cranford, T. W., Krysl, P. & Hildebrand, J. A. Acoustic pathways revealed: Simulated sound transmission and reception in Cuvier’s beaked whale (Ziphius cavirostris). *Bioinspir. Biomim.* **3**(1). 10.1088/1748-3182/3/1/016001 (2008). 10.1088/1748-3182/3/1/016001 18364560

[7] Cranford, T. W., Trijoulet, V., Smith, C. R. & Krysl, P. Validation of a vibroacoustic finite element model using bottlenose dolphin simulations: the dolphin biosonar beam is focused in stages. *Bioacoustics* **23**(2), 161–194. 10.1080/09524622.2013.843061 (2013).

[8] Dahlheim, M. E. *Bio-Acoustics of the Gray Whale (Eschrichtlus robustus)*. PhD thesis, University of British Columbia. 10.14288/1.0097975 (1987).

[9] Frankel, A. S. & Stein, P. J. Gray whales hear and respond to signals from a 21–25 kHz active sonar. *Mar. Mamm. Sci.* **36**(4), 1111–1125. 10.1111/mms.12700 (2020).

[10] Guazzo, R. A. et al. Migratory behavior of eastern North Pacific gray whales tracked using a hydrophone array. *PLoS ONE* **12**(10). 10.1371/journal.pone.0185585 (2017). 10.1371/journal.pone.0185585 PMC5662093 29084266

[11] Ketten, D. R. & Wartzok, D. Three-dimensional reconstructions of the dolphin EAR. In *Sensory Abilities of Cetaceans. NATO ASI Series* (Thomas, J. A. & Kastelein, R. A. eds.) Vol. 196, 81–105. 10.1007/978-1-4899-0858-2_6 (Springer, Boston, MA, 1990).

[12] Krysl, P. & Cranford, T. W. Animations of vibration modes obtained from finite element simulations performed on the skull of a juvenile gray whale [Dataset]. Dryad. 10.5061/dryad.dbrv15f8w (2024).

[13] Krysl, P., Cranford, T. W., Wiggins, S. M., & Hildebrand, J. A. Simulating the effect of high-intensity sound on cetaceans: Modeling approach and a case study for Cuvier’s beaked whale (*Ziphius cavirostris*). *J. Acoustical Soc. Am.* **120**(4), 2328–2339. 10.1121/1.2257988 (2006). 10.1121/1.2257988 17069328

[14] Krysl, P., Cranford, T. W. & Hildebrand, J. A. Lagrangian finite element treatment of transient vibration/acoustics of biosolids immersed in fluids. *Int. J. Numer. Meth. Eng.* **74(5)**, 754–775. 10.1002/nme.2192 (2007).

[15] Lancaster, W. C., Ary, W. J., Krysl, P. & Cranford, T. W. Precocial development within the tympanoperiotic complex in cetaceans. *Mar. Mamm. Sci.* **31**(1), 369–375. 10.1111/mms.12145 (2015).

[16] Sue E. Moore and Donald K. Ljungblad. Gray Whales in the Beaufort, Chukchi, and Bering Seas: Distribution and Sound Production. In *The Gray Whale: Eschrichtius Robustus* (Jones, M. L. et al. eds.) chapter 23, 543–559. 10.1016/B978-0-08-092372-7.50029-7 (Academic Press, 1984).

[17] Morris, M., Krysl, P., Hildebrand, J. & Cranford, T. Resonance of the tympanoperiotic complex of fin whales with implications for their low frequency hearing. *PLoS ONE* **18**(10), 1–20. 10.1371/journal.pone.0288119 (2023). 10.1371/journal.pone.0288119 PMC10566675 37819911

[18] Ridgway, S. H. & Carder, D. A. Assessing hearing and sound production in cetaceans not available for behavioral audiograms: Experiences with sperm, pygmy sperm, and gray whales. *Aquatic Mammals* **27**(3), 267–276 (2001).

[19] Soldevilla, M. S. et al. Cuvier’s beaked whale (*Ziphius cavirostris*) head tissues: Physical properties and CT imaging. *J. Exp. Biol.* **208**(12), 2319–2332. 10.1242/jeb.01624 (2005). 15939773 10.1242/jeb.01624

[20] Southall, B. L. et al. Data collection and analysis methods to evaluate potential impacts of seismic surveys and other marine industrial activities on baleen whales, *Ocean Coast. Manag.* 245. 10.1016/j.ocecoaman.2023.106799 (2023).

[21] Southall, B. L. et al. Managing human activity and marine mammals: A biologically based, relativistic risk assessment framework. *Front. Mar. Sci.* 10. 10.3389/fmars.2023.1090132 (2023).

[22] Tubelli, A. A., Zosuls, A., Ketten, D. R. & Mountain, D. C. A model and experimental approach to the middle ear transfer function related to hearing in the humpback whale (*Megaptera novaeangliae*). *J. Acoustical Soc. Am.* **144**(2), 525–535. 10.1121/1.5048421 (2018). 10.1121/1.5048421 30180668

